# Association among Fibrinolytic Proteins, Metabolic Syndrome Components, Insulin Secretion, and Resistance in Schoolchildren

**DOI:** 10.1155/2015/170987

**Published:** 2015-11-08

**Authors:** Jin-Shuen Chen, Chung-Ze Wu, Nain-Feng Chu, Li-Chien Chang, Dee Pei, Yuh-Feng Lin

**Affiliations:** ^1^Division of Nephrology, Department of Internal Medicine, Tri-Service General Hospital, National Defense Medical Center, Taipei 11490, Taiwan; ^2^Division of Endocrinology and Metabolism, Department of Internal Medicine, Shuang Ho Hospital, Taipei Medical University, New Taipei City 23561, Taiwan; ^3^Department of Internal Medicine, School of Medicine, College of Medicine, Taipei Medical University, Taipei 11031, Taiwan; ^4^Taitung Hospital, Ministry of Health and Welfare, Taitung County 95043, Taiwan; ^5^School of Public Health, National Defense Medical Center, Taipei 11490, Taiwan; ^6^School of Pharmacy, National Defense Medical Center, Taipei 11490, Taiwan; ^7^Division of Endocrinology and Metabolism, Department of Internal Medicine, Cardinal Tien Hospital, Xindian, New Taipei City 23148, Taiwan; ^8^Medical School, Catholic Fu Jen University, New Taipei City 24205, Taiwan; ^9^Graduate Institute of Clinical Medicine, College of Medicine, Taipei Medical University, Taipei 11031, Taiwan; ^10^Division of Nephrology, Department of Internal Medicine, Shuang Ho Hospital, Taipei Medical University, New Taipei City 23561, Taiwan

## Abstract

We investigated the role of urokinase plasminogen activator (uPA) and its soluble receptors (suPAR) and plasminogen activator inhibitor-1 (PAI-1) in metabolic syndrome (MetS) components, insulin secretion, and resistance in schoolchildren. We enrolled 387 children, aged 10.3 ± 1.5 years, in Taipei. Anthropometry, fibrinolytic proteins, MetS components, insulin secretion, and resistance were measured. Subjects were divided into normal, overweight, and obese groups. Finally, the relationship between fibrinolytic proteins and metabolic syndrome in boys and girls was analyzed. In boys, PAI-1 was positively associated with body mass index (BMI) percentile, hypertriglyceride, insulin secretion, and resistance. In girls, PAI-1 was positively associated with obesity, hypertriglyceridemia, and insulin secretion. In girls, uPA was positively associated with insulin secretion. suPAR was positively associated with high-sensitivity C-reactive protein in both boys and girls, and with BMI percentile and body fat in girls. The obese boys had higher suPAR and PAI-1 levels than the normal group. The obese girls had higher uPA, suPAR, and PAI-1 than the normal group. Boys and girls with MetS had higher PAI-1. Fibrinolytic proteins, especially PAI-1, are associated with MetS components and insulin secretion in children. Fibrinolytic proteins changes were more likely to occur in girls than in boys.

## 1. Introduction

Metabolic syndrome (MetS) started as a concept rather than a diagnosis [1]. Currently, the definition of MetS is a constellation of interconnected risk factors of metabolic origin that often accompany obesity, the principal symptom, and consist of dyslipidemia, elevated blood pressure, impaired glucose tolerance, a hypercoagulable state, and a proinflammatory state.

Childhood obesity is increasing and has become an important public health issue in the world. With an increase in prevalence of obesity in childhood, development of type 2 diabetes mellitus (DM) and cardiovascular disease is starting earlier. The prevalence of MetS has increased rapidly in schoolchildren [2]; therefore, there is a need to study the pathogenesis and morbidities of MetS earlier in childhood.

Urokinase plasminogen activator (uPA) and its receptors (uPAR) are well known as fibrinolytic proteins and commonly used for thrombolytic therapy in occlusive artery disease. Besides their functions in the fibrinolytic cascade, uPA and uPAR are expressed by a variety of hemopoietic cells [3] and upregulated during endotoxemia [4]. uPA and uPAR also participate in activation and proliferation of T lymphocytes [5, 6]. Accordingly, uPA and uPAR contribute to regulation of immune response and inflammation [7]. Furthermore, uPAR may be shed from the cell surface by several proteases as a soluble bioactive peptide in blood and body fluid, called soluble uPAR (suPAR). Compelling evidence shows that suPAR not only is correlated to inflammatory diseases and sepsis but also is a potent predictive biomarker for mortality in critically ill patients [8]. Moreover, uPAR participates in mechanism of extracellular matrix degradation and several cancer cells metastasis [9, 10]. On the other hand, suPAR could be a useful biomarker for predictive prognosis of some cancers [11, 12]. In recent studies, suPAR in adults has also been associated with and predictive of diseases including cardiovascular diseases, stroke, and DM [13–15]. It is a reasonable presumption that uPA and suPAR contribute to development of lifestyle-related insulin resistance and MetS. However, there is no study that shows that uPA/suPAR is associated with or contributes to development of insulin resistance and MetS, especially in children. Furthermore, plasminogen activator inhibitor-1 (PAI-1) not only is well recognized for regulating fibrinolysis by inhibiting plasmin activity but also is associated with atherothrombosis and MetS [16]. Thus, the role of these fibrinolytic proteins in insulin resistance is critical.

MetS may be preventable if we clearly understand its pathophysiology and intervene in lifestyle decisions early [17]. So far, there is no study surveying changes in uPA, suPAR, and PAI-1 in MetS, insulin secretion, and resistance, especially in children. In the present study, we explored the relationship among uPA, suPAR, PAI-1, and components of MetS in schoolchildren to determine the relationship among insulin secretion and resistance and changes of fibrinolytic proteins.

## 2. Methods

### 2.1. Subjects

The subjects, elementary Taipei schoolchildren aged 6 to 12 years, were enrolled in the study following their routine annual health exam. Subjects with major medical diseases, including hypertension, diabetes mellitus, heart disease, liver cirrhosis, renal failure, and other significant medical or surgical issues, were excluded. Subjects taking medications affecting insulin resistance or secretion were also excluded. In addition, subjects who refused to participate or had missing or incomplete data were excluded. The study was approved by the Institutional Review Board of our institution (approval number: TYGGH102021), and the nature, purpose, and potential risks of the study were explained to the subjects and their parents before obtaining their consent to participate.

### 2.2. Anthropometry and Laboratory Measurements

Participants completed the questionnaire about dietary patterns, lifestyle, and pubertal status (the development of penis/testes and pubic hair for boys and breast growth and pubic hair for girls) under the help of trained nurses. Anthropometric measurements, including body mass index (BMI), body fat, systolic blood pressure (SBP), and diastolic blood pressure (DBP), were assessed by trained research nurses. After fasting for 10–12 hours overnight, around 10 mL of venous blood samples was drawn from an antecubital vein by using venous containers for subsequent biochemical analysis. Body height was recorded to the nearest 0.1 cm while subjects were barefoot and wearing light indoor clothing. Body weight was recorded to the nearest 0.1 kg, and body fat (%) was measured by bioelectric impedance using a segmental body composition analyzer (Tanita Corp., Tokyo, Japan). BMI was calculated as weight in kilograms divided by the square of height in meters. Blood pressure was measured on the right arm after patients rested for 10 minutes in a sitting position, using an appropriately sized cuff of a HEM-740C automatic digital BP monitor (Omron Corp., Tokyo, Japan).

Plasma and serum were separated from blood within 1 hour and stored at −80°C until measurement. Serum concentrations of triglyceride (TG) were measured with randox reagent on a Hitachi 7150 autoanalyzer (Hitachi, Tokyo, Japan). High-density lipoprotein-cholesterol (HDL) and low-density lipoprotein-cholesterol (LDL) were measured enzymatically with Daiichi reagent on an Olympus AU600 autoanalyzer (Olympus, Tokyo, Japan). Plasma glucose was measured using the YSI 203 glucose oxidase analyzer (Yellow Spring Instrument, Yellow Spring, OH). Insulin was measured using the Coat-A-Count solid-phase radioimmunoassay kit (Diagnostic Products, Los Angeles, CA). High-sensitivity C-reactive protein (hsCRP) was measured using IMMULITE 2000 immunoassay system (Siemens, Los Angeles, CA). The human uPA, suPAR, and PAI-1 concentrations were measured by the Human ELISA kits (DY1310, DUO00, DSE100, R&D systems, Minneapolis, MN) in duplicate. The intra-assay and interassay coefficients of variation on uPA were 6.5% and 7.9%, respectively. The intra-assay and interassay coefficients of variation on suPAR were 4.1% and 5.1%, respectively. The intra-assay and interassay coefficients of variation on PAI-1 were 6.8% and 7.0%, respectively. Homeostasis model assessment-insulin resistance (HOMA-IR) and homeostasis model assessment-*β* (HOMA-*β*) were measured to assess insulin resistance and insulin secretion, respectively [18]. The formula of HOMA-IR and HOMA-*β* is shown as follows:

(1) HOMA-IR=fasting insulin×fasting glucose22.5,HOMA-β=20×fasting insulinfasting glucose−3.5.

### 2.3. Statistical Analysis

SPSS version 13.0 statistical package for Windows (SPSS, Chicago, IL) was used for data analysis. Subjects were divided into normal, overweight, and obese groups by the BMI percentile as defined by the Ministry of Health and Welfare standard in Taiwan (normal: 0–90%, overweight: 90.1~95%, and obese: >95%). Furthermore, development of MetS in boys and girls was assessed. The criteria of MetS in children were based on the modified definition of the International Diabetes Federation [19], with three or more characteristics of five criteria: BMI > 90th percentile, TG > 1.7 mmol/L, HDL < 1.03 mmol/L, blood pressure > 130 mmHg systolic or > 85 mmHg diastolic, and FPG > 5.6 mmol/L. The continuous variables were expressed as mean ± SD. The correlations between the uPA, suPAR, PAI-1, and components of MetS in different genders were evaluated with Pearson's correlation. One-way ANOVA with Bonferroni* post hoc* test was applied to compare the difference among normal, overweight, and obese groups in boys and girls. Independent student *t*-test was applied to difference of fibrinolytic proteins between children with MetS and without MetS. All statistical data were expressed as two-sided, and *P* values less than 0.05 were considered to be statistically significant.

## 3. Results

### 3.1. Relationship among uPA, suPAR, PAI-1, and Components of MetS, Insulin Resistance, and Secretion

A total of 361 schoolchildren (172 boys and 189 girls) participated in the study. The relationship among fibrinolytic proteins, components of MetS, insulin secretion, and resistance is shown in Table 1. In boys, the levels of uPA were not significantly related to age, components of MetS, hsCRP, insulin resistance, or insulin secretion, but suPAR levels were associated with hsCRP. Significantly, in boys, PAI-1 was positively related to BMI percentile, TG, HOMA-*β*, and HOMA-IR. In girls, uPA was positively related to HOMA-*β*. The suPAR levels in girls were positively related to age, BMI percentile, body fat, and hsCRP. The PAI-1 levels in girls were positively related to body fat, TG, and HOMA-*β*. Because insulin secretion, resistance, and MetS components vary with age and puberty, all variables were individually adjusted by age and pubertal stage. After adjusting for age, the uPA levels were still related to HOMA-*β* in girls (*r* = 0.178, *P* = 0.015). The suPAR levels were still significantly related to hsCRP in boys (*r* = 0.184, *P* = 0.016) and to BMI percentile (*r* = 0.188, *P* = 0.01), body fat (*r* = 0.196, *P* = 0.007), and hsCRP (*r* = 0.206, *P* = 0.004) in girls. The PAI-1 levels were still significantly related to BMI percentile (*r* = 0.185, *P* = 0.016), TG (*r* = 0.181, *P* = 0.018), HOMA-*β* (*r* = 0.249, *P* = 0.001), and HOMA-IR (*r* = 0.153, *P* = 0.045) in boys. However, the PAI-1 levels were significant to only TG (*r* = 0.159, *P* = 0.029) in girls after adjusting for age. The majority of children were prepubertal (stage I). Only a few girls (*n* = 7) were at pubertal stage II. After adjustment for pubertal stage, all results were similar.

### 3.2. uPA, suPAR, and PAI-1 in Normal, Overweight, and Obese Children

The demographic data among normal, overweight, and obese groups in different genders is shown in Table 2. Both obese boys and girls had more body fat, higher TG, hsCRP, and HOMA-*β*, and lower HDL than normal boys and girls. FPG, DBP, and LDL among normal, overweight, and obese groups showed no significant difference in either boys or girls. The difference between the two genders, however, is that obese boys had higher SBP and obese girls had more HOMA-IR.

**Figure 1:**
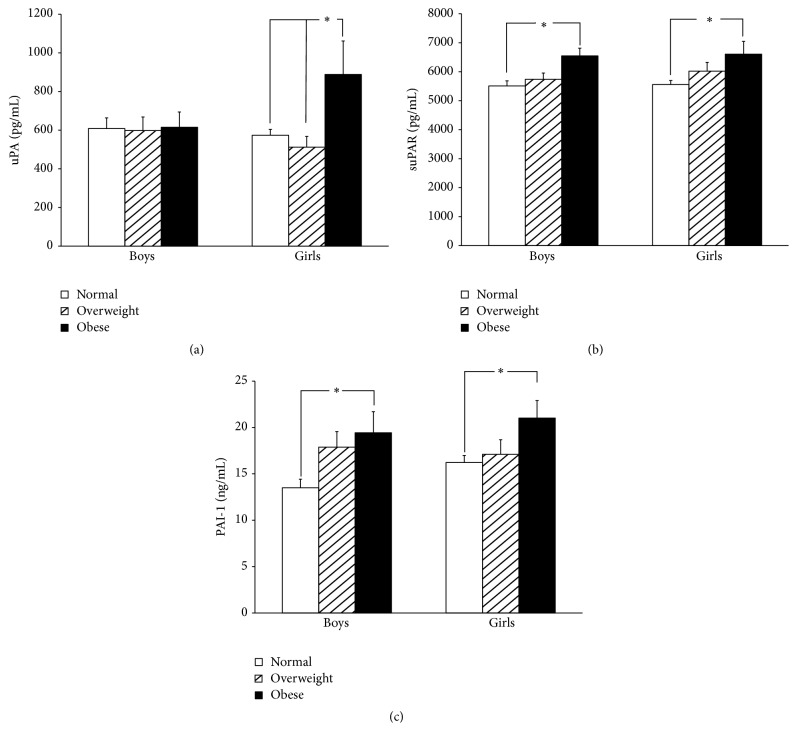
(a) Urokinase plasminogen activator (uPA), (b) soluble urokinase plasminogen activator receptor (suPAR), and (c) plasminogen activator inhibitor-1 (PAI-1) in normal, overweight, and obese children. The uPA levels in obese girls are higher than in overweight and normal girls. In addition, suPAR and PAI-1 levels in the obese children are higher than in the normal group in both genders. Mean ± SE; ^*∗*^ *P* < 0.05.

Comparisons of uPA, suPAR, and PAI-1 levels in normal, overweight, and obese children in different genders are shown in Table 2 and Figure 1. In general, obese boys had significantly higher suPAR (6546.0 ± 1679.5 pg/mL versus 5509.2 ± 1652.7 pg/mL; *P* = 0.003) and PAI-1 (19.43 ± 14.35 ng/mL versus 13.59 ± 8.62 ng/mL; *P* = 0.016) levels than normal boys. However, the uPA levels among the three groups of boys were similar (normal: 608.6 ± 516.5 pg/mL, overweight: 597.9 ± 468.5 pg/mL, and obese: 614.9 ± 502.8 pg/mL; *P* = 0.987). Clearly, suPAR and PAI-1 levels in girls increased with BMI percentage. The obese girls had significantly higher suPAR (6606.6 ± 2286.0 pg/mL versus 5558.0 ± 1604.9 pg/mL; *P* = 0.015) and PAI-1 (21.01 ± 9.83 ng/mL versus 16.22 ± 8.73 ng/mL; *P* = 0.038) than normal girls. It is noteworthy that uPA levels between normal and overweight girls were similar, but uPA levels abruptly and significantly increased in obese girls (888.2 ± 899.9 pg/mL versus 573.8 ± 343.9 pg/mL, 511.9 ± 324.5 pg/mL; *P* = 0.003).

### 3.3. uPA, suPAR, and PAI-1 in Children with and without MetS

**Figure 2:**
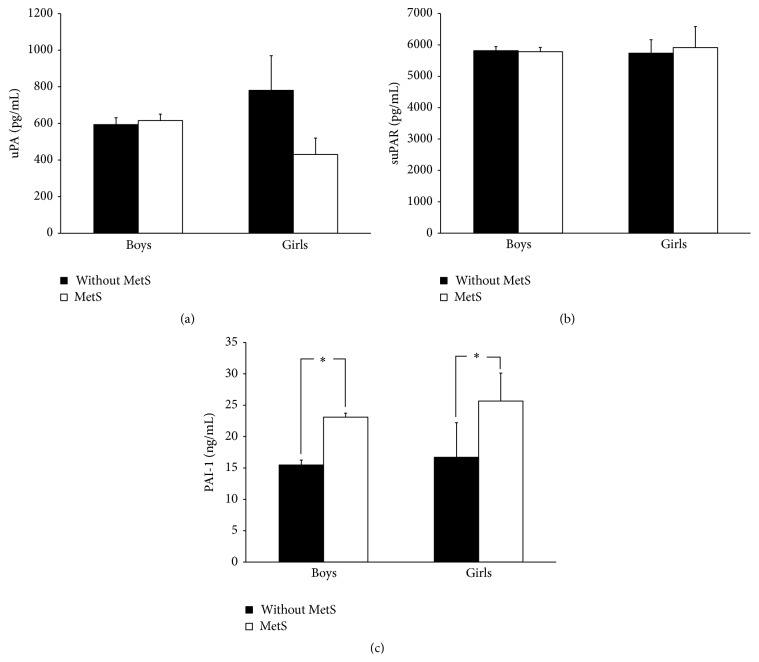
(a) Urokinase plasminogen activator (uPA), (b) soluble urokinase plasminogen activator receptor (suPAR), and (c) plasminogen activator inhibitor-1 (PAI-1) in children with and without metabolic syndrome (MetS). The uPA and suPAR levels are not significantly different between children with and without MetS. However, children with MetS have higher PAI-1 levels. Mean ± SE; ^*∗*^ *P* < 0.05.

There were 13 boys (7.55%) and 8 girls (4.23%) who fit the definition of MetS. The change of uPA, suPAR, and PAI-1 in boys and girls with or without MetS is shown in Figure 2. Generally, boys and girls with MetS had higher levels of PAI-1 (boys: 23.09 ± 19.86 ng/mL versus 15.50 ± 9.91 ng/mL; girls: 25.42 ± 12.65 ng/mL versus 16.70 ± 8.75 ng/mL). However, there was no significant difference in uPA (boys: 780.5 ± 683.8 pg/mL versus 593.1 ± 480.5 pg/mL; girls: 430.0 ± 254.0 pg/mL versus 615.4 ± 479.7 pg/mL) and suPAR (boys: 5734.8 ± 1553.4 pg/mL versus 5814.7 ± 1666.4 pg/mL; girls: 5915.2 ± 1895.2 pg/mL versus 5785.7 ± 1774.0 pg/mL) levels in children with and without MetS in both genders.

## 4. Discussion

The present study is the first paper exploring the relationship among fibrinolytic proteins, components of MetS, insulin secretion, and resistance in schoolchildren. In boys, PAI-1 was associated with obesity, hypertriglyceridemia, insulin secretion, and resistance. In girls, PAI-1 was associated with obesity, hypertriglyceridemia, and insulin secretion. In girls, uPA was associated with insulin secretion. Furthermore, suPAR was associated with inflammation in both boys and girls. In addition, suPAR was associated with obesity in girls. The obese boys had significantly higher suPAR and PAI-1 levels than normal boys. The obese girls had significantly higher uPA, suPAR, and PAI-1 than normal girls. Boys and girls with MetS had higher PAI-1.

Among fibrinolytic proteins, PAI-1 is one component associated with atherosclerosis. As early as 30 years ago, Vague and his colleague found that PAI-1 was linked to insulin resistance, insulin, and weight in obesity [20]. Moreover, PAI-1 levels significantly declined in patients after administration of Metformin for improving insulin resistance [21]. In recent years, more and more evidence has shown that PAI-1, an adipocytokine secreted by adipose tissue [22], is associated with MetS [16]. Not surprisingly, in our study, we found that circulating PAI-1 levels were associated with obesity, dyslipidemia, and insulin secretion in both genders. It is interesting that PAI-1 is positively related to blood pressure and insulin resistance in girls, but not in boys. In Western New York Study, Donahue et al. investigated 1,455 participants without DM or cardiovascular disease and found higher circulating PAI-1 levels only among prediabetic women [23]. Their results were similar to our findings that greater endothelial dysfunction and change of fibrinolytic proteins are more likely in females. But our findings showed that the association becomes apparent as early as childhood. However, more study is needed to determine causation.

Although uPA's thrombolytic function, converting plasminogen to plasmin, is well known, new evidence shows that uPA and its receptor contribute to the formation of atheroma by migration of and chemotaxis of macrophage [24]. However, few studies have explored the role of uPA in MetS. We found that uPA is not associated with any components of MetS in both genders. However, uPA is associated with insulin secretion in girls. In boys, the relation coefficient between uPA and HOMA-*β* increased but was not significant. However, it is noteworthy that the uPA levels in normal and overweight girls were similar, but uPA levels abruptly increased in obese girls. Whether obese girls with increasing uPA also have increased insulin secretion needs further study.

In recent years, suPAR is regarded as a candidate nontraditional biomarker for inflammation or infection and predicting prognosis of sepsis. Our results showed that suPAR is associated with hsCRP in both boys and girls. The relation between suPAR and inflammation found in our study is similar to that in previous studies. However, we found that suPAR is also associated with obesity in girls, not in boys. Some evidence shows that obesity is associated with subclinical inflammation [25]. According to our results, increasing suPAR is more likely to occur in obese girls. Similarly, Lyngbæk et al. found that suPAR is associated with BMI and waist circumference in nonsmoking women, not men [26]. The role of suPAR in different genders is still unclear. In addition, we also found that the suPAR is weakly negatively related to age in girls, not in boys. The result is contradictory to other studies in the adult population. The causation of relationship between suPAR and age in prepubertal girls is still unknown and needs a large-scale investigation and further study in the future.

Generally, the changes of fibrinolytic proteins in our findings were more prominent in obese girls than in obese boys, which seem to contradict previous clinical observation that young men had a higher rate of atherosclerosis and coronary artery disease than young women. Our explanation may be that girls are not prone to endothelial dysfunction in general. However, if they become obese, fibrinolytic proteins change is more likely than in their male counterparts.

There were some limitations in our study. First, the number of subjects was relatively small. Consequently, some variables showed tendencies but did not reach significant difference. Second, these children come from a small area of Taiwan. We did not use time- and cost-consuming stratified sampling to test children in the whole country.

In conclusion, PAI-1 is associated with obesity, hypertriglyceridemia, insulin secretion, and MetS and suPAR is associated with inflammation in children. Furthermore, fibrinolytic proteins, especially PAI-1, are associated with MetS components and insulin secretion varies with gender in children. This biochemical mechanism needs further exploration in future study.

## Figures and Tables

**Table 1:** Correlation among fibrinolytic proteins, metabolic syndrome components, and HOMA-*β* and HOMA-IR in boys and girls.

	Boys			Girls		
	uPA	suPAR	PAI-1	uPA	suPAR	PAI-1
Age	0.091	−0.120	0.092	−0.020	−0.151^*∗*^	0.137
BMI percentile	−0.065	0.072	0.189^*∗*^	0.084	0.174^*∗*^	0.118
Body fat	−0.043	0.130	0.029	0.100	0.157^*∗*^	0.146^*∗*^
FPG	0.037	−0.025	0.061	−0.014	−0.052	0.035
SBP	−0.102	0.104	0.089	0.087	0.056	0.132
DBP	0.038	−0.069	0.110	−0.042	−0.026	0.121
TG	−0.027	0.059	0.194^*∗*^	−0.010	−0.080	0.164^*∗*^
HDL	0.109	−0.116	−0.148	0.089	−0.068	−0.099
LDL	0.049	−0.075	0.128	0.031	−0.034	0.020
hsCRP	−0.040	0.180^*∗*^	−0.008	−0.018	0.225^*∗*^	−0.011
HOMA-*β*	0.124	0.127	0.254^*∗∗*^	0.167^*∗*^	0.021	0.159^*∗*^
HOMA-IR	0.096	0.074	0.164^*∗*^	0.081	−0.023	0.125

uPA: urokinase plasminogen activator, suPAR: soluble urokinase plasminogen activator receptor, PAI-1: plasminogen activator inhibitor-1, BMI: body mass index, FPG: fasting plasma glucose, SBP: systolic blood pressure, DBP: diastolic blood pressure, TG: triglyceride, HDL: high-density lipoprotein-cholesterol, LDL: low-density lipoprotein-cholesterol, HOMA-*β* (insulin secretion): homeostatic model assessment-*β*, and HOMA-IR (insulin resistance): homeostatic model assessment-insulin resistance.

^*∗*^<0.05;^*∗∗*^<0.01.

**Table 2:** General characteristics of components of metabolic syndrome, insulin secretion, and resistance in boys and girls.

	Boys			Girls		
	Normal	Overweight	Obese	Normal	Overweight	Obese
*n*	88	44	40	128	34	27
Age (year)	10.3 ± 1.6^†^	11.1 ± 1.3^*∗*,§^	9.7 ± 1.4^†^	10.1 ± 1.5	10.5 ± 1.4	10.3 ± 1.8
BMI percentile (%)	53.5 ± 23.8^†,§^	92.3 ± 2.6^*∗*^	97.6 ± 1.1^*∗*^	48.3 ± 24.1^†,§^	90.1 ± 2.7^*∗*^	97.3 ± 1.2^*∗*^
Body fat (%)	22.2 ± 6.4^§^	23.9 ± 3.3^§^	27.3 ± 2.8^*∗*,†^	19.7 ± 2.8^†,§^	25.0 ± 2.7^*∗*,§^	28.2 ± 1.9^*∗*,†^
FPG (mmol/L)	5.07 ± 0.42	5.12 ± 0.40	5.11 ± 0.43	4.93 ± 0.39	5.02 ± 0.31	4.96 ± 0.36
SBP (mmHg)	113.0 ± 9.0^†,§^	120.9 ± 9.7^*∗*^	122.2 ± 11.3^*∗*^	115.8 ± 10.2^†^	121.2 ± 9.1^*∗*^	120.9 ± 9.5
DBP (mmHg)	71.2 ± 7.8	73.2 ± 8.3	72.5 ± 9.4	73.8 ± 9.9	71.8 ± 6.2	75.2 ± 11.2
TG (mg/dL)	0.62 ± 0.27^†,§^	0.90 ± 0.49^*∗*^	0.96 ± 0.57^*∗*^	0.73 ± 0.30^§^	0.96 ± 0.38	1.31 ± 1.59^*∗*^
HDL (mg/dL)	1.47 ± 0.33^†,§^	1.29 ± 0.29^*∗*^	1.18 ± 0.27^*∗*^	1.42 ± 0.29^§^	1.30 ± 0.27	1.16 ± 0.33^*∗*^
LDL (mg/dL)	2.54 ± 0.68	2.43 ± 0.57	2.64 ± 0.59	2.40 ± 0.68	2.47 ± 0.50	2.64 ± 0.55
hsCRP	0.68 ± 1.07^§^	0.72 ± 0.84^§^	1.58 ± 1.45^*∗*,†^	0.43 ± 0.82^†,§^	1.03 ± 1.35^*∗*^	1.17 ± 1.13^*∗*^
HOMA-*β*	164.4 ± 164.3^§^	233.1 ± 178.8	253.2 ± 118.2^*∗*^	209.9 ± 218.4^§^	245.8 ± 214.6	362.0 ± 336.5^*∗*^
HOMA-IR	3.09 ± 3.95	4.40 ± 5.90	4.10 ± 2.35	3.34 ± 4.10^§^	4.24 ± 3.45	6.05 ± 5.37^*∗*^
uPA (pg/mL)	608.6 ± 516.5	597.9 ± 468.5	614.9 ± 502.8	573.8 ± 343.9^§^	511.9 ± 324.5^§^	888.2 ± 899.9^*∗*,†^
suPAR (pg/mL)	5509.3 ± 1652.7^§^	5737.3 ± 1449.1	6546.0 ± 1679.5^*∗*^	5558.0 ± 1604.9^§^	6021.7 ± 1758.6	6606.6 ± 2286.0^*∗*^
PAI-1 (ng/mL)	13.59 ± 8.62^§^	17.88 ± 11.12	19.43 ± 14.35^*∗*^	16.22 ± 8.73^§^	17.10 ± 9.21	21.01 ± 9.83^*∗*^

Data shown: mean ± SD, uPA: urokinase plasminogen activator, suPAR: soluble urokinase plasminogen activator receptor, PAI-1: plasminogen activator inhibitor-1, BMI: body mass index, FPG: fasting plasma glucose, SBP: systolic blood pressure, DBP: diastolic blood pressure, TG: triglyceride, HDL: high-density lipoprotein-cholesterol, LDL: low-density lipoprotein-cholesterol, HOMA-*β* (insulin secretion): homeostatic model assessment-*β*, and HOMA-IR (insulin resistance): homeostatic model assessment-insulin resistance; ^*∗*^ *P* < 0.05 against normal, ^†^ *P* < 0.05 against overweight, and ^§^ *P* < 0.05 against obese.
